# Sleep trajectories and osteoporosis incidence: findings from two prospective cohort studies

**DOI:** 10.3389/fpubh.2025.1654798

**Published:** 2025-10-07

**Authors:** Xiangxiang Zhang, Zongshan Li, Huanyong Tian, Tian Lv, JinXiang Shang, Ermin Cai

**Affiliations:** ^1^Shaoxing Seventh People's Hospital (Affiliated Mental Health Center, Medical College of Shaoxing University, Shaoxing, Zhejiang, China; ^2^Department of Neurology, Xuanwu Hospital, Capital Medical University, Beijing, China; ^3^Department of Radiotherapy, Zhuji Affiliated Hospital of Wenzhou Medical University, Zhuji, Zhejiang, China; ^4^Department of Neurology, Zhuji Affiliated Hospital of Wenzhou Medical University, Zhuji, Zhejiang, China; ^5^Department of Orthopedics, The Affiliated Hospital of Shaoxing University, Shaoxing City, Zhejiang, China; ^6^Shaoxing Key Laboratory of Precision Diagnosis and Treatment for Osteoporosis, Zhuji, Zhejiang, China; ^7^Department of Radiology, Zhuji Affiliated Hospital of Wenzhou Medical University, Shaoxing, Zhejiang, China

**Keywords:** sleep quality, trajectories, osteoporosis, longitudinal cohort, public health

## Abstract

**Background:**

Most studies evaluate sleep quality at a single time point, and few have employed repeated measurements to investigate this association. This longitudinal research investigated changes in sleep quality patterns among older adults and examined their relationship with the onset of osteoporosis.

**Methods:**

We analyzed data from two prospective cohorts: Participants comprised 4,328 individuals from the English Longitudinal Study of Aging (ELSA) and 9,132 from the U.S. Health and Retirement Study (HRS). Sleep quality was quantified using standardized sleep quality scores, and trajectories were determined based on baseline and follow-up assessments. Changes in sleep quality status were categorized to reflect persistent, improving, or deteriorating patterns. Associations between sleep quality trajectories and osteoporosis incidence were examined using Cox proportional hazards regression models.

**Results:**

At baseline, sleep quality was significantly associated with the prevalence of osteoporosis in both datasets (ELSA: HR = 1.11, 95% CI: 1.08–1.15; HRS: HR = 1.1, 95% CI: 1.07–1.13). During the follow-up period, compared with participants with persistently good sleep quality, those with persistently poor sleep quality had a significantly increased risk of osteoporosis (ELSA: HR = 1.89, 95% CI: 1.47–2.44; HRS: HR = 1.52, 95% CI: 1.26–1.82).

**Conclusion:**

Poor sleep trajectories significantly increase osteoporosis risk, suggesting sleep improvement may help prevent bone loss. These consistent findings across two cohorts support sleep-focused interventions as a potential osteoporosis prevention strategy.

## Introduction

As a prevalent skeletal disorder, osteoporosis involves diminished bone mineral content, structural breakdown of trabecular bone, and imbalances in bone metabolism (1, 2). Fundamentally, it stems from various etiologies that disrupt normal bone metabolism, resulting in bone resorption exceeding bone formation (3). Although it can affect individuals at any age, osteoporosis is particularly common among postmenopausal women and older men (4, 5). This pathological condition increases susceptibility to fragility fractures, especially in load-bearing bones, and the associated complications substantially elevate mortality and long-term disability risks in aging populations (6). As global aging accelerates, the incidence of osteoporosis is rising rapidly, posing a significant public health challenge.

Lifestyle factors such as diet and exercise are well-established determinants of osteoporosis risk. Adequate intake of calcium and vitamin D supports bone mineralization, while weight-bearing and resistance exercises stimulate bone remodeling and help preserve bone density (7).

Sleep quality is a critical determinant of population health and serves as a key indicator of whether an individual's sleep is restorative (8). High-quality sleep facilitates recovery by replenishing energy and promoting cellular repair processes (9). It exerts widespread influence across physiological systems, notably the metabolic system (10). However, poor sleep quality is increasingly prevalent. Epidemiological data estimate that the prevalence of sleep disorders among adults is ~30%−40% (11). Contributing factors such as the accelerated pace of life, increased occupational stress, and pervasive use of electronic devices have led to a rise in sleep disturbances, particularly among urban residents, older adults, and individuals with chronic illnesses (12, 13). Enhancing sleep quality is thus vital for health maintenance and may mitigate the risk of numerous diseases.

Previous studies have shown that poor sleep quality is associated with a higher risk of developing osteoporosis (14). However, these investigations typically rely on sleep quality assessments at a single time point, overlooking dynamic changes over time. In contrast, longitudinal assessments of sleep quality can better capture its evolving biological implications, including its relationship with osteoporosis progression. Importantly, accumulating evidence indicates that sleep quality can be improved through targeted interventions (15, 16). Evaluating the association between changes in sleep quality and the risk of osteoporosis may yield valuable insights into the potential of sleep-focused strategies for the prevention and management of this condition. Therefore, there is an urgent need to examine how changes in sleep quality influence the risk of developing osteoporosis. In this study, we leverage data from two large-scale prospective cohorts—the English Longitudinal Study of Aging (ELSA) and the Health and Retirement Study (HRS). By analyzing trajectories of sleep quality change, we aim to elucidate their association with osteoporosis incidence and provide evidence to inform public health interventions.

## Materials and methods

### Study population

This prospective study utilized data from the English Longitudinal Study of Aging (ELSA) and the Health and Retirement Study (HRS) (17, 18). Both datasets provided detailed information on demographics, socioeconomic status, health conditions, and blood-based laboratory measures (19). Participants from ELSA were selected from Wave 6 (2012–2013), with Wave 10 (2020–2021) serving as the final follow-up. Similarly, HRS participants were drawn from Wave 12 (2014–2015), with the final follow-up data available through Wave 15 (2020–2021). Individuals with complete baseline sleep quality data were considered for inclusion.

**Figure 1:**
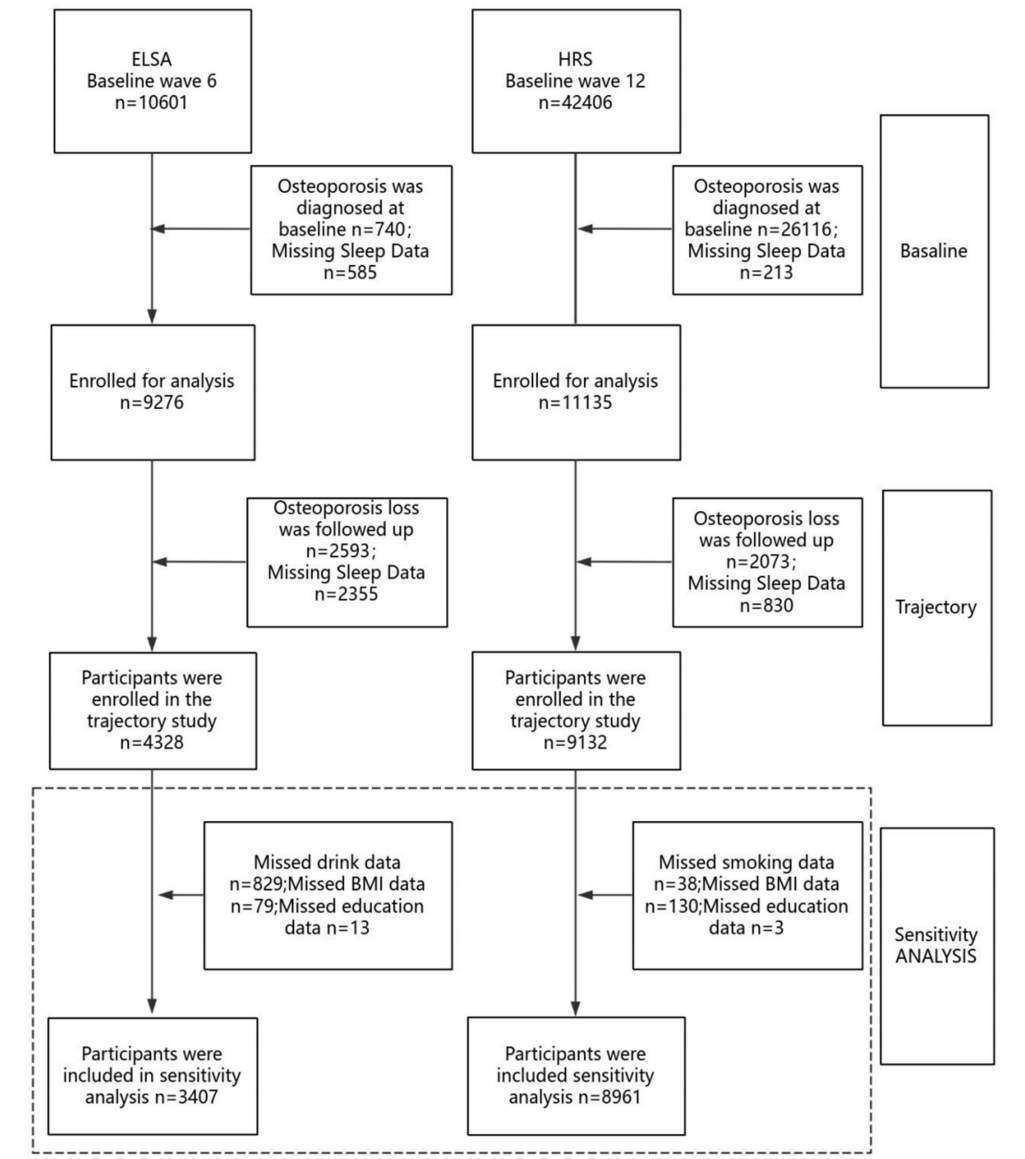
Flow chart of the study population.

Participants were excluded based on the following criteria: (1) diagnosis of osteoporosis at baseline; (2) missing baseline sleep quality data; and (3) missing data on osteoporosis or sleep quality during follow-up, as illustrated in Figure 1. *ELSA cohort*: Of the 10,601 participants initially assessed for baseline sleep quality, exclusions were made for baseline osteoporosis diagnosis (*n* = 740), missing baseline sleep quality data (*n* = 585), missing follow-up data on osteoporosis (*n* = 2,593), and missing follow-up sleep quality data (*n* = 2,355). A total of 4,328 participants were ultimately included in the final analysis. *HRS cohort*: Among 42,406 participants initially assessed for baseline sleep quality, exclusions were applied for baseline osteoporosis diagnosis and missing questionnaire data (*n* = 26,016), missing baseline sleep quality data (*n* = 213), missing follow-up data on osteoporosis (*n* = 2,073), and missing follow-up sleep quality data (*n* = 830). The final analytic sample included 9,132 participants. The specific procedures are shown in Figure 1.

To avoid heterogeneity due to variation in disease duration, participants with a diagnosis of osteoporosis at baseline were excluded. Thus, only incident cases of osteoporosis during follow-up were considered in the analysis.

### Sleep quality assessment

*ELSA Cohort*: Sleep quality served as a key variable in this study and was measured using four items adapted from the Jenkins Sleep Scale: (1) frequency of difficulty falling asleep; (2) frequent awakenings during the night; (3) waking up feeling fatigued and exhausted; and (4) a self-reported global assessment of overall sleep quality. The Jenkins Sleep Scale has been previously validated in both clinical populations and large-scale cohort studies (20). For the first three items, participants reported the frequency of each sleep problem over the past month, with response options as follows: (1) not at all (score = 1); (2) less than once per week (score = 2); (3) once or twice per week (score = 3); and (4) three or more times per week (score = 4). For the fourth item, participants rated their overall sleep quality as: (1) very good (score = 1); (2) good (score = 2); (3) fairly poor (score = 3); and (4) very poor (score = 4). An overall sleep quality score was derived by calculating the mean of the four item scores. A total score was calculated by summing responses to all three items (range: 4–16), with scores of 4–11 classified as good sleep quality and 12–16 as poor sleep quality (21). *HRS Cohort*: Four items assessing the type and frequency of sleep disturbances, adapted from the Jenkins Sleep Scale, were included in the physical health section of the Health and Retirement Study (HRS) (22). The items were: (1) “How often do you have trouble falling asleep?” (2) “How often do you wake up during the night?” (3) “How often do you wake up too early and find yourself unable to fall back asleep?” and (4) “When you wake up in the morning, how often do you feel well-rested?” Response options for each item were: rarely or never (scored = 1), sometimes (scored = 2), and most of the time (scored = 3). A total sleep quality score was calculated by summing the scores across all four items, yielding a range from 4 to 12. Scores from 4 to 8 indicated good sleep quality, while scores from 9 to 12 indicated poor sleep quality (23).

Based on changes in sleep quality between baseline and final assessment, participants were categorized into three trajectory groups: (1) maintained: no change in sleep quality classification; (2) improved: transitioned from poor to good sleep quality; (3) worsened: transitioned from good to poor sleep quality (23, 24).

### Osteoporosis assessment

The assessment of osteoporosis in both the ELSA and HRS cohorts was primarily based on self-reported physician diagnoses. During the structured interviews, participants were inquired, “Have you ever been diagnosed with osteoporosis by a physician?” Those who answered “yes” were categorized as having osteoporosis (25, 26). In the ELSA cohort, the incidence of osteoporosis was monitored biennially from Wave 6 (2012–2013) through Wave 10 (2020–2021). In the HRS cohort, data collection began in Wave 12 (2014–2015) and continued through Wave 15 (2020–2021).

### Covariates

Covariates considered in this study included demographic characteristics, baseline clinical data, and comorbidities. Demographic variables were categorized by age, sex (male or female), marital status (divorced, married, or never married), and educational attainment (non-college vs. college). Baseline clinical information included smoking and alcohol consumption, both assessed through self-report and dichotomized as “yes” or “no.” Body mass index (BMI) was calculated in kg/m^2^ and used to classify obesity status. Information on comorbidities was obtained by asking participants whether they had ever been diagnosed with conditions such as diabetes or hypertension. The choice of these variables was guided by prior research that investigated the clinical significance of sleep quality within the ELSA and HRS datasets (27).

### Statistical analysis

Baseline sleep quality and its association with osteoporosis risk were analyzed, with continuous variables represented by means and standard errors, and categorical variables described using frequency distributions and percentages (28, 29). Analysis of variance (ANOVA) was employed to assess differences in continuous variables across groups, whereas categorical variables were evaluated using chi-square tests (30, 31). Each participant's follow-up time was calculated from baseline until the date of osteoporosis diagnosis or the end of the study period, whichever came first. Cox proportional hazards regression models were employed to estimate hazard ratios (HRs) and 95% confidence intervals (CIs) for associations between sleep quality, sleep duration, and osteoporosis risk. Three regression models were constructed: Model 1: adjusted for Sleep quality score; Model 2: adjusted for Sleep quality score, age, sex, education, and marital status; Model 3: additionally adjusted for body mass index, diabetes mellitus, hypertension, smoking status, and alcohol consumption. Missing covariate data were imputed using multiple imputation (32).

Only through the use of restricted cubic spline regression in the fully adjusted model could the potential non-linear relationship between sleep quality scores and the risk of osteoporosis be thoroughly explored (33).

We conducted a sensitivity analysis to address potential bias arising from missing covariate data by excluding participants with incomplete information and re-evaluating the association between sleep quality and osteoporosis.

## Results

### Baseline characteristics of the study population

The final sample for baseline osteoporosis assessment included 4,328 participants from ELSA (mean age 63.77, 55.15% women) and 9,132 participants from HRS (mean age 64.99, 55.45% women), as determined by the inclusion and exclusion criteria. A description of participants' baseline characteristics can be found in Table 1. Participants diagnosed with osteoporosis tended to be older, predominantly female, less frequently married or partnered, and reported lower educational attainment and physical activity levels compared to those without the condition. In addition, participants with osteoporosis had lower body mass index (BMI) and were more likely to consume alcohol than those without osteoporosis. Because participants with baseline osteoporosis were excluded, all osteoporosis cases reported in this study represent incident diagnoses identified during follow-up.

**Table 1:** Baseline analysis of osteoporosis status of participants' baseline characteristics.

	**ELSA**				**HRS**			
	**Total (*****n*** = **4,328)**	**Non- osteoporosis (*****n*** = **3,879)**	**Osteoporosis (*****n*** = **449)**	***P*** **-value**	**Total (*****n*** = **9,132)**	**Non-osteoporosis (*****n*** = **8,029)**	**Osteoporosis (*****n*** = **1,103)**	***P*** **-value**
Age	63.77 ± 7.96	63.34 ± 7.78	67.55 ± 8.49	< 0.0001	64.99 ± 9.67	64.63 ± 9.59	67.60 ± 9.85	< 0.0001
**Sex**								
Female	2,387 (55.15)	2,035 (52.46)	352 (78.40)	< 0.0001	5,064 (55.45)	4,165 (51.87)	899 (81.50)	< 0.0001
Male	1,941 (44.85)	1,844 (47.54)	97 (21.60)		4,068 (44.55)	3,864 (48.13)	204 (18.50)	
**Marital**								
Divorced	792 (18.30)	663 (17.09)	129 (28.73)	< 0.0001	2,397 (26.25)	2,019 (25.15)	378 (34.27)	< 0.0001
Married	3,319 (76.69)	3,024 (77.96)	295 (65.70)		6,289 (68.87)	5,606 (69.82)	683 (61.92)	
Never married	217 (5.01)	192 (4.95)	25 (5.57)		446 (4.88)	404 (5.03)	42 (3.81)	
**Education**								
Non-college	2,148 (49.63)	1,876 (48.36)	272 (60.58)	< 0.0001	4,234 (46.36)	3,728 (46.43)	506 (45.87)	0.75
College	2,180 (50.37)	2,003 (51.64)	177 (39.42)		4,898 (53.64)	4,301 (53.57)	597 (54.13)	
**BMI**								
< 25	1,148 (26.52)	997 (25.70)	151 (33.63)	< 0.01	2,115 (23.16)	1,775 (22.11)	340 (30.83)	< 0.0001
>30	1,335 (30.85)	1,203 (31.01)	132 (29.40)		3,434 (37.60)	3,042 (37.89)	392 (35.54)	
25–30	1,845 (42.63)	1,679 (43.28)	166 (36.97)		3,583 (39.24)	3,212 (40.00)	371 (33.64)	
**Smoke**								
No	3,662 (84.61)	3,286 (84.71)	376 (83.74)	0.64	4,997 (54.72)	4,456 (55.50)	541 (49.05)	< 0.0001
Yes	666 (15.39)	593 (15.29)	73 (16.26)		4,135 (45.28)	3,573 (44.50)	562 (50.95)	
**Drink**								
Yes	3,938 (90.99)	3,553 (91.60)	385 (85.75)	< 0.0001	5,450 (59.68)	4,886 (60.85)	564 (51.13)	< 0.0001
No	390 (9.01)	326 (8.40)	64 (14.25)		3,682 (40.32)	3,143 (39.15)	539 (48.87)	
**Diabetes**								
No	4,006 (92.56)	3,596 (92.70)	410 (91.31)	0.33	6,989 (76.53)	6,133 (76.39)	856 (77.61)	0.39
Yes	322 (7.44)	283 (7.30)	39 (8.69)		2,143 (23.47)	1,896 (23.61)	247 (22.39)	
**Hypertension**								
No	2,195 (50.72)	1,984 (51.15)	211 (46.99)	0.11	3,871 (42.39)	3,419 (42.58)	452 (40.98)	0.33
Yes	2,133 (49.28)	1,895 (48.85)	238 (53.01)		5,261 (57.61)	4,610 (57.42)	651 (59.02)	
Baseline JSS score^*^	8.79 ± 3.10	8.68 ± 3.06	9.82 ± 3.28	< 0.0001	6.62 ± 2.03	6.57 ± 2.01	7.00 ± 2.10	< 0.0001
**Baseline sleep quality classification**								
Good	3,424 (79.28)	3,123 (80.66)	301 (67.34)	< 0.0001	7,506 (82.19)	6,655 (82.89)	851 (77.15)	< 0.0001
Poor	895 (20.72)	749 (19.34)	146 (32.66)		1,626 (17.81)	1,374 (17.11)	252 (22.85)	
**Follow-up sleep quality classification**								
Good	3,435 (79.44)	3,113 (80.34)	322 (71.71)	< 0.0001	7,508 (82.22)	6,654 (82.87)	854 (77.43)	< 0.0001
Poor	889 (20.56)	762 (19.66)	127 (28.29)		1,624 (17.78)	1,375 (17.13)	249 (22.57)	
**Sleep quality trajectory**								
Maintaining good quality group	3,050 (70.68)	2,796 (72.29)	254 (56.82)	< 0.0001	6,738 (73.78)	6,001 (74.74)	737 (66.82)	< 0.0001
Maintaining poor quality group	515 (11.94)	435 (11.25)	80 (17.90)		856 (9.37)	721 (8.98)	135 (12.24)	
Quality improved group	377 (8.74)	311 (8.04)	66 (14.77)		770 (8.43)	653 (8.13)	117 (10.61)	
Quality worsened group	373 (8.64)	326 (8.43)	47 (10.51)		768 (8.41)	654 (8.15)	114 (10.34)	

JSS^*^ score, ELSA: 4–16, HRS: 4–12.

ELSA, English Longitudinal Study of Aging; HRS, U.S. Health and Retirement Study; BMI, body mass index; JSS, Jenkins Sleep Scale.

### Association between baseline sleep quality and osteoporosis

The total sleep quality scores of participants from ELSA and HRS were analyzed using both continuous and categorized representations. When analyzed as a continuous variable in ELSA, sleep quality score was positively associated with osteoporosis. In Model 1, HR = 1.11, 95% CI: 1.08–1.15, *P* < 0.0001. After adjustment in Model 2, HR = 1.09, 95% CI: 1.06–1.12, *P* < 0.0001. Further adjustment in Model 3 still showed a positive association (HR = 1.11, 95% CI: 1.08–1.15, *P* < 0.0001). When sleep quality was categorized, individuals reporting poor sleep demonstrated a significantly elevated osteoporosis risk compared to their counterparts with good sleep quality. In Model 1, HR = 1.94 (95% CI: 1.59–2.36, *P* < 0.0001); in Model 2, HR = 1.69 (95% CI: 1.38–2.06, *P* < 0.001); and in Model 3, HR = 1.92 (95% CI: 1.57–2.35, *P* < 0.0001).

When HRS sleep quality scores were analyzed as continuous variables, a positive association with osteoporosis was also observed. In Model 1, HR = 1.1, 95% CI: 1.07–1.13, *P* < 0.0001. In Model 2, HR = 1.08, 95% CI: 1.05–1.12, *P* < 0.0001. Model 3 continued to show a positive association (HR = 1.1, 95% CI: 1.07–1.13, *P* < 0.0001).

When sleep quality was analyzed as a categorical variable, participants with poor sleep quality showed a significantly increased risk of developing osteoporosis compared to those with good sleep quality. In Model 1, the hazard ratio (HR) was 1.40 (95% CI: 1.22–1.62; *P* < 0.0001); in Model 2, the HR was 1.33 (95% CI: 1.15–1.53; *P* < 0.001); and in Model 3, the HR increased to 1.41 (95% CI: 1.22–1.62; *P* < 0.0001). These findings suggest a robust and consistent association between poor baseline sleep quality and a higher risk of osteoporosis across all adjustment models. Detailed results are presented in Table 2.

**Table 2:** Association between sleep quality and osteoporosis at baseline.

**Sleep quality:good**	**Model 1**		**Model 2**		**Model 3**	
**Sleep quality**	**95% CI**	***P*** **-value**	**95% CI**	***P*** **-value**	**95% CI**	***P*** **-value**
**Good**	**Ref**		**Ref**		**Ref**	
**ELSA**						
Poor	1.94 (1.59, 2.36)	< 0.0001	1.69 (1.38, 2.06)	< 0.0001	1.92 (1.57, 2.35)	< 0.0001
Sleep quality score	1.11 (1.08, 1.15)	< 0.0001	1.09 (1.06, 1.12)	< 0.0001	1.11 (1.08, 1.15)	< 0.0001
**HRS**						
Poor	1.4 (1.22, 1.62)	< 0.0001	1.33 (1.15, 1.53)	< 0.0001	1.41 (1.22, 1.62)	< 0.0001
Sleep quality score	1.1 (1.07, 1.13)	< 0.0001	1.08 (1.05, 1.12)	< 0.0001	1.1 (1.07, 1.13)	< 0.0001

Model 1: Sleep quality/sleep quality score.

Model 2: Sleep quality/sleep quality score, age, sex, education, marital.

Model 3: Sleep quality/sleep quality score, age, sex, education, marital, body mass index, diabetes, hypertension, smoke, drink.

**Figure 2:**
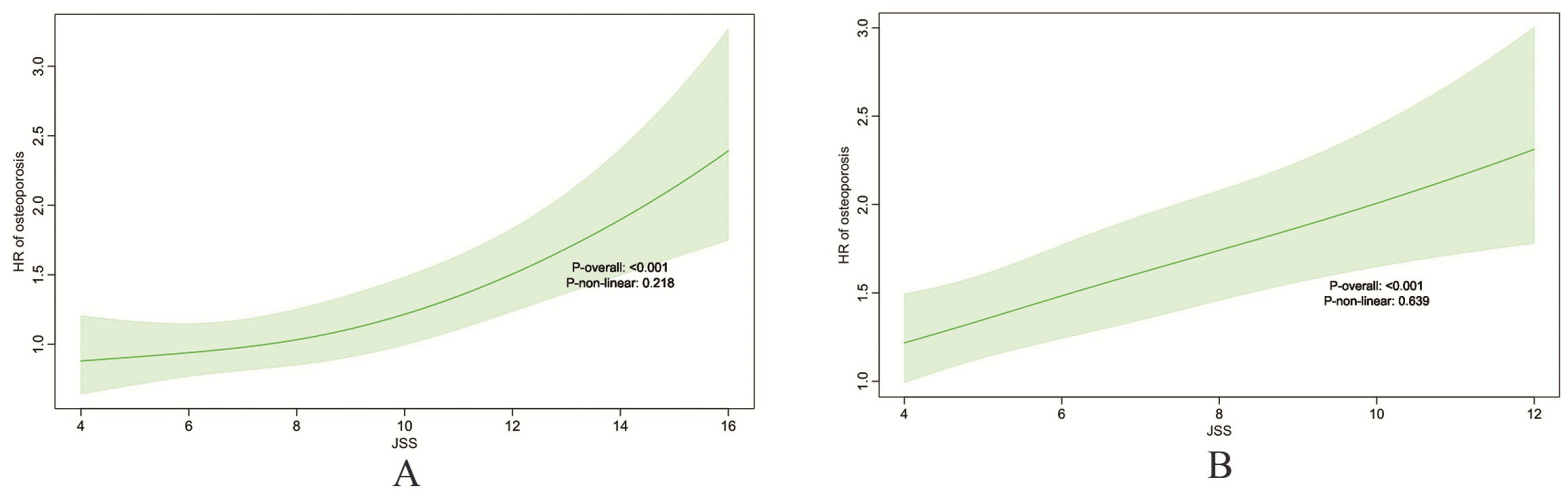
Restricted cubic spline (RCS) plots illustrating the association between baseline sleep quality score and osteoporosis risk. ELSA **(A)**, HRS **(B)**.

As shown in Figure 2, there is a clear relationship between baseline sleep quality scores and osteoporosis risk.

### Association between sleep quality changes and osteoporosis

Supplementary Table 1 presents the number and percentage of participants who experienced changes in sleep quality over the 2-year follow-up period. Among those with good sleep quality at baseline, 373 participants (8.64%) in the ELSA group and 768 participants (8.41%) in the HRS group experienced a deterioration to poor sleep quality. In contrast, among those with poor sleep quality at baseline, 377 participants (8.74%) in the ELSA group and 770 participants (8.43%) in the HRS group showed improvement to good sleep quality.

Table 3 presents the associations between changes in sleep quality and osteoporosis risk. Compared with participants with stable good sleep quality, those with persistently poor sleep quality had a higher risk of developing osteoporosis (ELSA, HR = 1.89, 95% CI: 1.47–2.44; HRS, HR = 1.52, 95% CI: 1.26–1.82). Among participants with poor baseline sleep quality who improved to good sleep quality, the risk of osteoporosis remained significantly elevated compared to those with stable good sleep (ELSA, HR = 2.25, 95% CI: 1.71–2.95; HRS, HR = 1.42, 95% CI: 1.16–1.73). In contrast, participants whose sleep quality deteriorated from good to poor also had a significantly increased risk of osteoporosis (ELSA, HR = 1.54, 95% CI: 1.13–2.11; HRS, HR = 1.44, 95% CI: 1.18–1.75).

**Table 3:** Association between changes in sleep quality and the risk of osteoporosis.

**Maintaining good quality group**	**Model 1**		**Model 2**		**Model 3**	
**Character**	**95% CI**	***P*** **-value**	**95% CI**	***P*** **-value**	**95% CI**	***P*** **-value**
**Maintaining good quality group**	**Ref**		**Ref**		**Ref**	
**ELSA**						
Maintaining poor quality group	1.94 (1.51, 2.50)	< 0.0001	1.61 (1.25, 2.08)	< 0.001	1.89 (1.47, 2.44)	< 0.0001
Quality improved group	2.21 (1.68, 2.89)	< 0.0001	1.98 (1.50, 2.60)	< 0.0001	2.25 (1.71, 2.95)	< 0.0001
Quality worsened group	1.54 (1.13, 2.11)	0.01	1.33 (0.97, 1.82)	0.07	1.54 (1.13, 2.11)	0.01
*P* for trend		< 0.0001		< 0.0001		< 0.0001
**HRS**						
Maintaining poor quality group	1.49 (1.24, 1.79)	< 0.0001	1.43 (1.19, 1.72)	< 0.001	1.52 (1.26, 1.82)	< 0.0001
Quality improved group	1.43 (1.17, 1.73)	< 0.001	1.33 (1.10, 1.62)	0.004	1.42 (1.16, 1.73)	< 0.001
Quality worsened group	1.39 (1.14, 1.70)	0.001	1.37 (1.13, 1.67)	0.002	1.44 (1.18, 1.75)	< 0.001
*P* for trend		< 0.0001		< 0.0001		< 0.0001

Model 1: Sleep quality trajectories.

Model 2: Sleep quality trajectories, age, sex, education, marital.

Model 3: Sleep quality trajectories, age, sex, education, marital, body mass index, diabetes, hypertension, smoke, drink.

### Sensitivity analysis

We conducted a sensitivity analysis by reassessing the changes in sleep quality status. Consistent results were observed in both the ELSA and HRS cohorts (see Supplementary Tables 2, 3), supporting a persistent and significant association between worsening sleep quality and an increased risk of osteoporosis.

### Subgroup analysis related to osteoporosis association

To explore potential cohort-specific differences in the sleep quality–osteoporosis association, we conducted further analyses separately within the ELSA and HRS populations, we conducted stratified subgroup analyses based on age group (≥60 vs. < 60), sex, education level (low or high), smoking status (no, former, or current), alcohol consumption (heavy, low, or current), diabetes status (yes or no), and hypertension (yes or no). *ELSA cohort*: Stratified analysis revealed significant interactions in sex and smoking status (*P* for interaction = 0.008 and < 0.001, respectively), suggesting that these two factors may enhance the impact of sleep quality on osteoporosis. Interactions with other variables, such as age, education level, alcohol consumption, diabetes, and hypertension, were not significant, indicating a certain degree of robustness of this association across different populations. *HRS cohort*: In all subgroups, poor sleep quality was significantly associated with an increased risk of osteoporosis. Interaction analysis showed a significant difference in age (*P* for interaction = 0.044), and near-significant interactions in sex (*P* = 0.073), education level (*P* = 0.096), and alcohol consumption (*P* = 0.092), suggesting that these factors may partially modulate the association. Interactions with smoking, diabetes, and hypertension status were not significant (*P* > 0.05), indicating that the effect of sleep quality on osteoporosis is relatively consistent across these groups. For details, refer to Supplementary Figure 1.

## Discussion

In this prospective cohort study, we identified distinct patterns in sleep quality and examined their associations with the risk of osteoporosis among middle-aged and older adults in the UK and US. As far as we are aware, no prior longitudinal studies have investigated how changes in sleep quality may influence the development or progression of osteoporosis. Our findings revealed that individuals with persistently poor sleep quality had a significantly increased risk of developing osteoporosis compared to those with consistently good sleep, underscoring the predictive value of sleep quality and its potential role in public health strategies for aging populations.

Previous studies examining the association between sleep quality and osteoporosis have been largely cross-sectional or limited to baseline assessments, failing to capture the dynamic nature of both sleep patterns and bone health. For example, Lee et al. (34) using data from 12,793 NHANES participants, reported an odds ratio of 5.57 (95% CI: 1.60–19.41) for osteoporosis in individuals with poor sleep quality, suggesting a substantial impact on bone mineral density. Similarly, Sasaki et al. (35) found a significant association between baseline PSQI scores and osteoporosis (β = 0.053, *P* < 0.05) among 1,032 adults aged 25–85. Bevilacqua et al. (36) also reported that perceived sleep quality was associated with changes in bone mineral density and microarchitecture in a cohort of 443 older adults. However, these studies did not account for changes in sleep quality over time, a critical limitation given the individualized and progressive nature of osteoporosis. By leveraging repeated assessments, our study offers a more comprehensive understanding of the temporal association between sleep quality and osteoporosis development.

A single baseline measure of sleep may not adequately reflect its long-term impact on bone health. Our findings emphasize the significance of sleep quality transitions. Compared to individuals with consistently good sleep, those whose sleep quality deteriorated over time exhibited a markedly higher risk of osteoporosis, suggesting that declining sleep quality may function as an independent risk factor. Participants with persistently poor sleep showed the highest risk, implying a cumulative detrimental effect of chronic sleep disturbances on bone metabolism. Notably, even participants whose sleep improved from poor to good still exhibited an elevated risk of osteoporosis, suggesting that previous periods of poor sleep may have lasting adverse effects, and that the benefits of sleep improvement on bone health may be delayed or only partially reversible.

These results highlight the importance of maintaining good sleep quality as a potential strategy to mitigate osteoporosis risk and support the integration of sleep assessment and intervention into preventive care, especially for aging populations. Although the biological mechanisms underlying the relationship between sleep disorders and osteoporosis are not fully elucidated, several plausible pathways have been proposed. First, As a consequence of chronic sleep disturbances, the hypothalamic–pituitary–adrenal (HPA) axis may become overactivated, thereby increasing circulating levels of ACTH and cortisol (37, 38). Elevated cortisol inhibits the differentiation of bone marrow stromal cells and enhances osteoclastic bone resorption, ultimately reducing bone formation (39). It also decreases calcium reabsorption in the renal tubules, contributing to an imbalance in bone remodeling that favors resorption over formation. Over time, these changes can result in decreased bone mineral density and increased osteoporosis risk. Second, sleep disorders are commonly associated with metabolic dysregulation, particularly hyperglycemia and insulin resistance (40). By impairing osteoblast differentiation, promoting apoptosis in bone-forming cells, and reducing circulating osteocalcin levels, hyperglycemia disrupts bone formation and increases susceptibility to osteoporosis (41). Third, poor sleep quality may contribute to bone loss through inflammatory mechanisms (42). Chronic sleep deprivation is linked to elevated inflammatory markers, which play key roles in bone remodeling and resorption (43, 44). Evidence suggests that inflammation, especially in individuals with shortened sleep duration, may mediate the deleterious effects of sleep disturbance on bone health (45). In addition, sleep disorders may reduce physical activity, depriving bones of necessary mechanical loading for remodeling. In addition to their direct effects, these conditions may contribute to poor nutrition and musculoskeletal deterioration—including low calcium and vitamin D intake, weight loss, and sarcopenia—which together intensify bone loss and increase osteoporosis susceptibility (46).

This study has important clinical and public health implications. First, our findings support the need for routine sleep quality assessments in middle-aged and older adults. We advocate for incorporating sleep quality evaluations into osteoporosis prevention and management strategies, recognizing sleep health as a vital component of geriatric care. Second, our results suggest that patients should be stratified based on sleep quality, with targeted early interventions—such as behavioral therapy, lifestyle modifications, and pharmacological treatment where appropriate—for those experiencing poor sleep. These interventions may help slow bone loss and reduce fracture risk. Furthermore, given the multifactorial etiology and dynamic progression of osteoporosis, individuals diagnosed with the condition should receive comprehensive, multimodal interventions rather than pharmacological treatment alone, in order to delay disease progression and potentially restore bone mass.

### Strengths

This study has several notable strengths. First, it is based on large, nationally representative cohorts, offering strong external validity and sufficient statistical power. Second, unlike most prior research limited to single time-point assessments, this study used repeated measures of sleep quality to characterize longitudinal trajectories, providing a more comprehensive understanding of sleep changes over time. Third, we investigated the temporal association between sleep quality trajectories and osteoporosis risk, adjusting for a wide range of covariates, thereby minimizing confounding and enhancing the validity of our findings.

### Limitations

However, several limitations should be acknowledged. First, as with most longitudinal studies, differential follow-up durations and attrition may have led to missing or unbalanced data. Second, although we adjusted for multiple potential confounders, the ELSA and HRS datasets lacked detailed information on some relevant factors such as genetic predisposition and the use of medications known to directly affect bone mineral density (e.g., glucocorticoids, antiepileptics, thyroid hormone replacement), which may have introduced residual confounding. Third, Sleep quality was assessed by self-report, which risks recall bias and prevents differentiation between specific sleep disorders such as insomnia or sleep apnea that may affect bone metabolism through distinct mechanisms. Fourth, the ELSA and HRS cohorts used slightly different sleep quality scoring systems, which may introduce measurement variability; however, consistent associations observed in cohort-specific analyses support the robustness of our findings. Fifth, Osteoporosis in this study was assessed through self-reported physician diagnoses rather than objective BMD. While this method has been widely applied in large-scale cohort studies (26), it may have led to misclassification and bias in our findings. Future research incorporating DXA-based BMD measures is warranted to confirm our results. Finally, Since we excluded baseline osteoporosis cases, all incident diagnoses represent new-onset disease during follow-up, and we could not account for pre-existing disease duration. Caution is warranted when generalizing these findings to younger populations or cross-cultural contexts, particularly given heterogeneity between UK/US cohorts in lifestyle and environmental factors.

## Conclusion

Using longitudinal data from ELSA and HRS, this study found that poor sleep quality is significantly associated with increased osteoporosis risk in middle-aged and older adults. Elevated risk was observed not only in those with persistently poor or worsening sleep, but also in those whose sleep improved over time. Maintaining healthy sleep patterns over time, as shown in this research, significantly contributes to better bone health outcomes. At the public health level, promoting awareness of sleep's impact on skeletal health and integrating sleep education into chronic disease prevention strategies for older adults may help mitigate osteoporosis risk.

## Data Availability

The original contributions presented in the study are included in the article/Supplementary material, further inquiries can be directed to the corresponding author.

## Supplementary material

Supplementary Figure 1 Stratified analyses of the association between sleep quality and osteoporosis.

Supplementary Table 1 Changes in sleep quality and percentages.

Supplementary Table 2 Association between sleep quality and osteoporosis in baseline sensitivity analysis.

Supplementary Table 3 Association between changes in sleep quality and risk of osteoporosis in sensitivity analysis.
