# Supplementary figures and images for: Sleep trajectories and osteoporosis incidence: findings from two prospective cohort studies
Source: Front Public Health. 2025 Oct 7;13:1654798. doi: 10.3389/fpubh.2025.1654798 (PMC12537439; doi:10.3389/fpubh.2025.1654798)

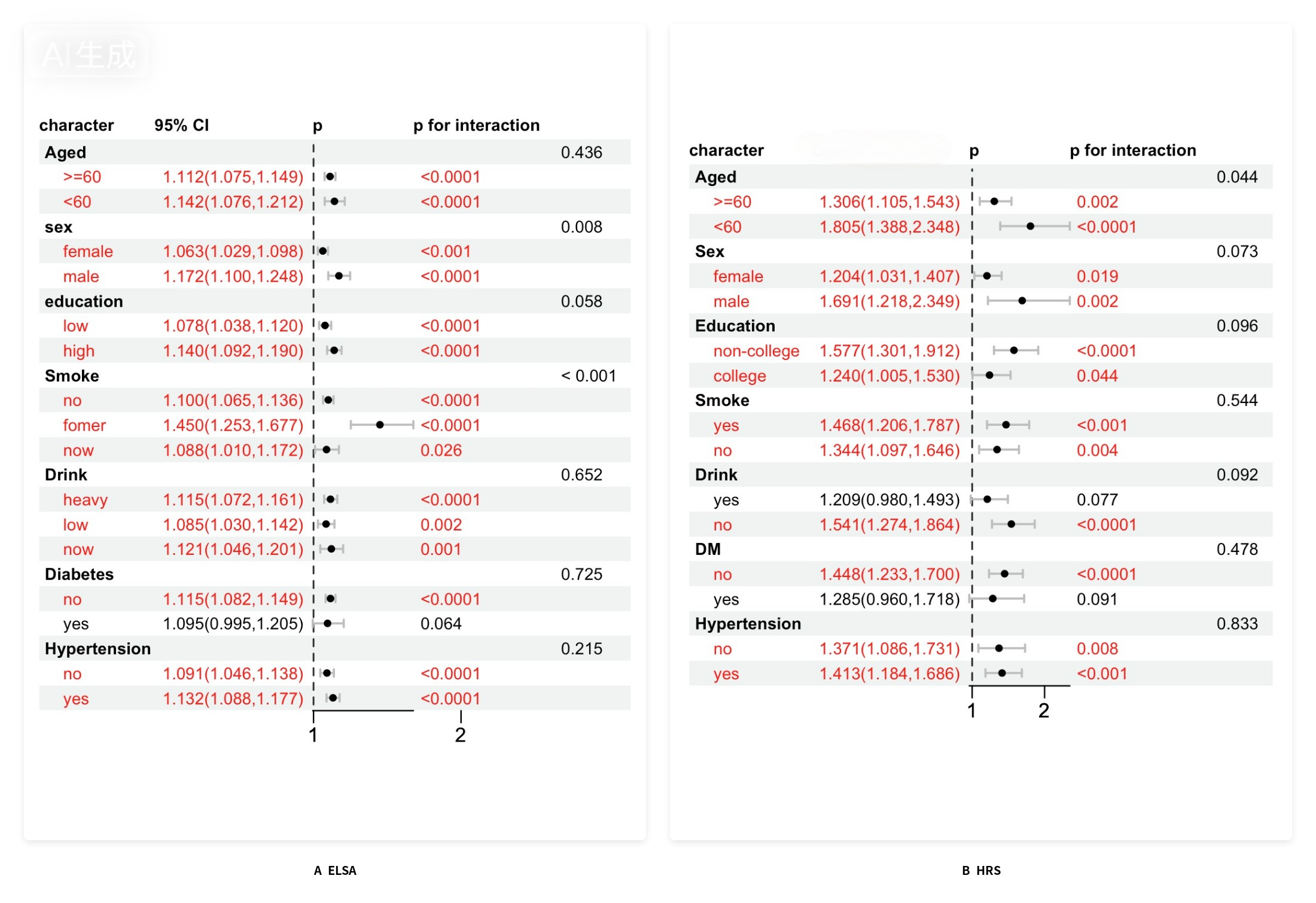

Supplement: Supplementary Figure 1 — Stratified analyses of the association between sleep quality and osteoporosis. [file Image_1.png]
