# Supplementary material for: Sleep trajectories and osteoporosis incidence: findings from two prospective cohort studies
Source: Front Public Health. 2025 Oct 7;13:1654798. doi: 10.3389/fpubh.2025.1654798 (PMC12537439; doi:10.3389/fpubh.2025.1654798)
Supplement: Supplementary Table 1 — Changes in sleep quality and percentages. [file Data_Sheet_1.doc]

| Supplementary Table 1: Changes in Sleep Quality and Percentages | | | |  |
| --- | --- | --- | --- | --- |
|  |
|  |  | ELSA | HRS |  |
| |  | | --- | | Maintaining good quality group | 3050(70.68) | 6738(73.78) |  |
| Sleep quality is good at baseline |  |  |  |  |
|  | Quality worsened group | 373( 8.64) | 768( 8.41) |  |
|  |  |  |  |  |
| |  | | --- | | Quality improved group | 377( 8.74) | 770( 8.43) |  |
| Sleep quality is poor at baseline |  |  |  |  |
|  | Maintaining poor quality group | 515(11.94) | 856( 9.37) |  |
