# Supplementary material for: Sleep trajectories and osteoporosis incidence: findings from two prospective cohort studies
Source: Front Public Health. 2025 Oct 7;13:1654798. doi: 10.3389/fpubh.2025.1654798 (PMC12537439; doi:10.3389/fpubh.2025.1654798)
Supplement: Supplementary Table 2 — Association between sleep quality and osteoporosis in baseline sensitivity analysis. [file Data_Sheet_2.doc]

| Supplementary Table 2：Association Between Sleep Quality and Osteoporosis in Baseline Sensitivity Analysis. | | | | | | |  |
| --- | --- | --- | --- | --- | --- | --- | --- |
|  |
|  | model 1 | | Model 2 | | Model 3 | |  |
| **ELSA** | | | | | | |  |
|  | 95%CI | P | 95%CI | P | 95%CI | P |  |
| Good | ref |  | ref |  | ref |  |  |
| Poor | 1.96(1.57,2.45) | <0.0001 | 1.68(1.34,2.11) | <0.0001 | 1.7(1.35,2.15) | <0.0001 |  |
| Sleep quality score | 1.11(1.08,1.15) | <0.0001 | 1.08(1.05,1.12) | <0.0001 | 1.09(1.05,1.13) | <0.0001 |  |
| **HRS** | | | | | | |  |
|  | 95%CI | P | 95%CI | P | 95%CI | P |  |
| Good | ref |  | ref |  | ref |  |  |
| Poor | 1.4(1.22,1.62) | <0.0001 | 1.33(1.15,1.53) | <0.0001 | 1.34(1.16,1.54) | <0.0001 |  |
| Sleep quality score | 1.1(1.07,1.13) | <0.0001 | 1.08(1.05,1.12) | <0.0001 | 1.09(1.06,1.12) | <0.0001 |  |
|  |  |  |  |  |  |  |  |
| model 1: Sleep quality/Sleep quality score | |  |  |  |  |  |  |
| model 2: Sleep quality/Sleep quality score, age, sex, education, marital | | | |  |  |  |  |
| model 3:Sleep quality/Sleep quality score, age, sex, education, marital, BMI, Diabetes, Hypertension, Smoke, drink | | | | | | |  |
