# Supplementary material for: Sleep trajectories and osteoporosis incidence: findings from two prospective cohort studies
Source: Front Public Health. 2025 Oct 7;13:1654798. doi: 10.3389/fpubh.2025.1654798 (PMC12537439; doi:10.3389/fpubh.2025.1654798)
Supplement: Supplementary Table 3 — Association between changes in sleep quality and risk of osteoporosis in sensitivity analysis. [file Data_Sheet_3.doc]

| Supplementary Table 3:Association Between Changes in Sleep Quality and Risk of Osteoporosis in Sensitivity Analysis | | | | | | |  |
| --- | --- | --- | --- | --- | --- | --- | --- |
|  |
| **ELSA s** | | | | | | |  |
|  | model 1 | | Model 2 | | Model 3 | |  |
| character | 95%CI | P | 95%CI | P | 95%CI | P |  |
| Maintaining good quality group | ref |  | ref |  | ref |  |  |
| Maintaining poor quality group | 1.92(1.45,2.56) | <0.0001 | 1.58(1.18,2.11) | 0.002 | 1.61(1.20,2.15) | 0.001 |  |
| Quality improved group | 2.24(1.65,3.03) | <0.0001 | 1.95(1.44,2.66) | <0.0001 | 2(1.47,2.72) | <0.0001 |  |
| Quality worsened group | 1.46(1.02,2.09) | 0.04 | 1.25(0.87,1.80) | 0.22 | 1.27(0.88,1.82) | 0.20 |  |
| p for trend |  | <0.0001 |  | <0.001 |  | <0.0001 |  |
| **HRS** | | | | | | |  |
|  | model 1 | | Model 2 | | Model 3 | |  |
| character | 95%CI | P | 95%CI | P | 95%CI | P |  |
| Maintaining good quality group | ref |  | ref |  | ref |  |  |
| Maintaining poor quality group | 1.49(1.24,1.79) | <0.0001 | 1.42(1.18,1.71) | <0.001 | 1.43(1.19,1.73) | <0.001 |  |
| Quality improved group | 1.43(1.17,1.73) | <0.001 | 1.33(1.10,1.62) | 0.004 | 1.35(1.11,1.64) | 0.003 |  |
| Quality worsened group | 1.39(1.14,1.70) | 0.001 | 1.36(1.12,1.66) | 0.002 | 1.37(1.13,1.68) | 0.002 |  |
| p for trend |  | <0.0001 |  | <0.0001 |  | <0.0001 |  |
|  |  |  |  |  |  |  |  |
| **sleepd** |  |  |  |  |  |  |  |
| model 1: Sleep quality trajectories |  |  |  |  |  |  |  |
| model 2: Sleep quality trajectories, age, sex, education, marital | | |  |  |  |  |  |
| model 3: Sleep quality trajectories , age, sex, education, marital,Body mass index, Diabetes, Hypertension, Smoke, Drink | | | | | | |  |
